# Impact of Mildly Elevated Alanine Transaminase on In‐Hospital Outcomes and Statin Intolerance in Elderly Patients With Acute Myocardial Infarction: A Retrospective Cohort Study

**DOI:** 10.1155/crp/5570312

**Published:** 2025-12-29

**Authors:** Yingcong Liang, Mingmin Li, Jiaxi Huang, Rui Wang, Yujing Mo, Xuyu He, Ling Xue

**Affiliations:** ^1^ Department of Cardiology, Guangdong Provincial People’s Hospital, Guangdong Academy of Medical Sciences, Southern Medical University, Guangzhou, China, fimmu.com; ^2^ Guangdong Cardiovascular Institute, Guangdong Provincial People’s Hospital, Guangdong Academy of Medical Sciences, Guangzhou, China, gdghospital.org.cn

**Keywords:** acute coronary syndrome, acute liver damage, cardiac critical care, geriatric cardiology, management of dyslipidemia

## Abstract

**Background:**

Mild alanine transaminase (ALT) elevation is common in older patients with acute myocardial infarction (AMI), but its prognostic value and implications for statin therapy remain unclear.

**Methods:**

This retrospective cohort study included 321 AMI patients aged ≥ 75 years admitted from 2014 to 2019 at Guangdong Provincial People’s Hospital. Mild ALT elevation was defined as ALT between the upper limit of normal (ULN) and 3 × ULN, and significant elevation as ALT > 3 × ULN. Patients were grouped by admission ALT into normal (N, *n* = 201), mildly elevated (ME, *n* = 104), and significantly elevated (SE, *n* = 16). Logistic regression analyses in SPSS 26.0 and R 3.4.3 assessed the association between ALT levels and in‐hospital mortality, adjusting for cardiac function, infarct size, renal function, and treatment factors.

**Results:**

Among survivors with elevated ALT, 87.4% achieved normalization before discharge. Statin intolerance was identified in 58 patients (18.9%) at admission and persisted in 6.7% at discharge. The ALT‐ME group had significantly higher statin intolerance (36.5% vs. 2.0%, *p* < 0.001) and higher in‐hospital mortality (18.3% vs. 6.0%, *p* = 0.001) compared with the ALT‐N group. Logistic regression analysis demonstrated that ALT elevation was independently associated with higher in‐hospital mortality (per 10 U/L ALT elevation, odds ratio 1.164, p = 0.010).

**Conclusion:**

In older patients with AMI, mild elevation in ALT levels upon admission is associated with worse in‐hospital outcomes, and statin intolerance is common and mostly reversible. Short‐term substitutes for statins should be considered in these patients.

## 1. Introduction

Coronary artery disease (CAD) remains the leading cause of death worldwide, accounting for 20.5 million deaths in 2021, with ischemic heart disease as the predominant contributor [1]. Without proper secondary prevention measures, patients with acute coronary syndrome, especially acute myocardial infarction (AMI), are at a considerable risk of recurrent episodes, leading to increased mortality. Statins remain the first‐line option for primary and secondary CAD prevention [2, 3]. The primary purpose of statin therapy is to control low‐density lipoprotein cholesterol (LDL‐C) levels and stabilize atherosclerotic plaques. Patients with AMI can benefit from early statin use, which is associated with a significantly lower in‐hospital mortality [4].

Overall, statin therapy is safe, with a low prevalence of statin intolerance [5]. However, there are safety concerns regarding statin use, including myopathy, liver dysfunction, diabetes mellitus, and drug–drug interactions [6]. Statins occasionally cause transaminase elevation; however, severe statin‐related liver damage is rare. Liver enzymes such as alanine aminotransferase (ALT), aspartate aminotransferase (AST), and alkaline phosphatase (ALP) have been identified as prognostic markers for mortality in various diseases. Ebrahimi et al. demonstrated their predictive value in COVID‐19 outcomes [7]. Among these enzymes, ALT is not influenced by skeletal muscle or myocardial injury, making it a specific marker of hepatocellular dysfunction in AMI patients receiving statins. In clinical practice, statin therapy is usually discontinued when ALT exceeds 3× the upper limit of normal (ULN) [8].

Some patients with AMI are at higher risk of liver dysfunction, including those with heart failure and advanced age, making early statin therapy less safe in these patients than in the general population [9]. Patients over 75 years of age are at higher risk of hepatic and renal impairment and more susceptible to adverse drug events during acute illnesses. As such, medication safety must be carefully considered in this population, particularly regarding statin use [10]. During AMI, these older patients face an increased risk of complications and may have reduced tolerance to statin therapy in the acute phase [11]. Previous studies have demonstrated a significant difference in prognosis depending on the degree of ALT elevation [8, 12, 13]. Li et al. found that in AMI patients, the in‐hospital mortality increased markedly only when ALT exceeded 3 × ULN (17.5% for ALT between ULN–2 × ULN, 24.3% for 2 × ULN–3 × ULN, and 53.8% for > 3 × ULN) [8]. In lipid management guidelines, this cutoff is also used to consider temporary discontinuation of statins, as supported by the GREACE study, which showed that statins remained beneficial and safe in patients with ALT < 3 × ULN [14]. However, the impact of advanced age and statin therapy has not been well explored, and detailed information on in‐hospital cardiovascular outcomes is lacking.

Currently, emerging lipid‐lowering agents, such as proprotein convertase subtilisin/kexin type 9 (PCSK9) inhibitors and ezetimibe, are alternative choice for lipid control in patients with AMI. Whether clinicians should use nonstatin lipid‐lowering agents as initial lipid control therapy in AMI patients at high risk of statin intolerance is still not clear, and no published clinical studies on AMI patients focused on dynamic change in ALT level during hospitalization and its relation with statin intolerance. To answer these clinical questions, we designed this retrospective cohort study in older (age ≥ 75 years) patients with AMI to investigate the relation among mild ALT elevation, statin therapy, and early prognosis.

## 2. Material and Methods

### 2.1. Study Population

In this retrospective cohort study, older patients aged ≥ 75 years diagnosed with AMI and admitted to the Cardiac Care Unit (CCU) of Guangdong Provincial People’s Hospital from January 2014 to December 2019 were consecutively enrolled. Since this study aimed to evaluate the impact of early ALT elevation on in‐hospital outcomes and statin use, limiting the cohort to CCU admissions helped ensure more consistent timing from AMI onset to ALT measurement and reduced variability due to delayed presentation. We predefined 75 years as the age cutoff for elderly patients, based on established international lipid management guidelines and prior evidence indicating increased vulnerability and underuse of high‐intensity statin therapy in this age group [15, 16]. The myocardial infarction (MI) diagnostic criteria were consistent with Type 1 MI in the fourth universal definition of MI, and the AMI onset was required to be within 7 days prior to admission [17]. Patients with ST‐segment elevation MI (STEMI) and non‐ST‐segment elevation MI (NSTEMI) were included. Participants with a history of hepatic disease, primarily chronic viral hepatitis, or malignant tumors were excluded. Hepatic disease was excluded based on documented past medical history. Patients with a history of chronic viral hepatitis (HBV or HCV) or liver malignancies were excluded. However, patients with transient transaminase elevation, hepatic hemangioma, or mild fatty liver detected on ultrasound were not excluded, as these conditions are common and not indicative of significant chronic liver disease. This study was approved by The Institutional Review Board of Guangdong Provincial People’s Hospital.

### 2.2. Data Collection

The information analyzed in this study was collected from electronic medical records by experienced data inspectors. Baseline demographic information and smoking status were collected from these medical records. The types of MI (STEMI or NSTEMI) were based on medical record diagnoses and reviewed by data inspectors based on electrocardiogram criteria. Medical history was based on standardized definitions, including hypertension, diabetes mellitus, prior CAD or MI, and prior stroke. We recorded vital signs and MI‐related information, including Killip class, symptom onset‐to‐balloon time, and coronary angiography information upon admission. Echocardiography was performed within 3 days of MI onset. The left ventricular ejection fraction (LVEF), measured using Simpson’s method, and right ventricular systolic dysfunction were used for further analysis.

Laboratory samples were collected for clinical purposes and analyzed (in accordance with the ISO 9000 Quality Management and Assurance Standards) using standard examination methods. The ULN for ALT was defined as 50 U/L for men and 40 U/L for women according to the recommended local guidelines. The reference intervals for ALT, AST, and GGT were defined according to the Chinese national guideline WS/T 404.1‐2012. This guideline adopted sex‐specific ULN values based on the 2010 multicenter IFCC study, which included Chinese population data [18]. Coagulopathy was defined as the lowest international normalized ratio (INR) within 3 days of admission > 1.20, to exclude the effect of heparin during percutaneous coronary intervention (PCI). The Chinese version of the modification of diet in renal disease equation was used to calculate the estimated glomerular filtration rate (eGFR) in this study.

All patients with AMI who underwent primary PCI received 20 mg atorvastatin before the intervention. Statin use upon admission was defined as long‐term prescription of statins within 48 h after PCI. Information on long‐term statin use by AMI survivors at discharge was recorded. All decisions regarding statin use were made by clinicians based on comprehensive clinical information, and participants who failed to tolerate statin therapy within 72 h after admission were defined as statin intolerant in this study, which is in line with the international definition [19].

Based on previous epidemiological data, the overall in‐hospital mortality in patients aged ≥ 75 years with AMI is estimated at approximately 10%. For this study, we assumed an 8% mortality rate in the ALT‐N group and 20% in the ALT‐ME group. Using an alpha level of 0.05 and a statistical power of 80%, the required sample size was calculated to be 204 patients. We then performed continuous retrospective screening of eligible cases to meet this sample size requirement and ultimately included 321 patients.

### 2.3. Statin Intolerance Definition and Assessment

Statin intolerance was defined according to the 2022 National Lipid Association (NLA) Scientific Statement on complete statin intolerance [20], which requires that a patient is unable to tolerate any statin at any dose. In this study, statin intolerance was adjudicated retrospectively based on clinical documentation and treatment decisions at two standardized timepoints: Within 48 h after PCI and within 72 h before hospital discharge. These timepoints were chosen to align with the availability of laboratory results and final prescribing information.

Patients were considered statin intolerant if they met at least one of the following criteria during either assessment window: (1) a prior diagnosis of statin intolerance; (2) presence of significant muscle‐related symptoms; (3) new ALT elevation after statin initiation with clinical deterioration risk; or (4) drug–drug interaction risk judged to be significant (e.g., use of amiodarone or other interacting medications).

To ensure consistency, we included only patients who were classified as completely statin intolerant, defined as those who did not receive any statin at any dose during the evaluation period.

### 2.4. Outcomes

The primary outcome of this study was in‐hospital all‐cause mortality. The secondary outcomes included mechanical complications of AMI, cardiogenic shock, severe ventricular arrhythmia, ischemic stroke, bleeding events, and in‐hospital ALT elevation. Mechanical complications of AMI were defined as papillary muscle rupture, ventricular free wall rupture, or ventricular septal rupture occurring during the index hospitalization. Cardiogenic shock was defined as clinical evidence of hypoperfusion with cardiac dysfunction, indicated by a cardiac index < 2.2 L/min/m^2^ or a LVEF < 50% during hospitalization. Severe ventricular arrhythmia was defined as ventricular fibrillation, polymorphic ventricular tachycardia, or sustained ventricular tachycardia lasting ≥ 30 s and requiring cardioversion; arrhythmias that occurred during primary PCI were excluded. Ischemic stroke was defined as new‐onset focal neurological symptoms during hospitalization confirmed by imaging evidence of cerebral ischemia or major intracranial or cervical arterial obstruction. Bleeding events were categorized using the Thrombolysis in Myocardial Infarction (TIMI) criteria: major bleeding referred to intracranial bleeding or clinically evident bleeding (including radiologic findings) with a hemoglobin drop ≥ 5 g/dL; minor bleeding was defined as clinically evident bleeding with a 3–5 g/dL hemoglobin drop; and minimal bleeding was defined as clinically evident bleeding with a hemoglobin drop < 3 g/dL. Coagulopathy was defined as an INR ≥ 1.20 at any point during hospitalization, excluding patients receiving anticoagulants such as heparin or warfarin. In‐hospital ALT elevation was defined as ALT elevation > 1 ULN within 7 days from the baseline level at admission; baseline ALT was taken from tests performed within 12 h before or 24 h after hospital admission.

### 2.5. Statistical Analyses

After testing for normality using the Kolmogorov–Smirnov test, continuous data were summarized as the mean ± standard deviation or median (interquartile range). Mann–Whitney or unpaired *t*‐tests were used for comparison between groups. Pearson’s chi‐square or Fisher’s exact tests were used for categorical data, as appropriate. Based on the ALT level upon admission, participants were categorized into three groups: ALT normal (ALT ≤ ULN, ALT‐N group), ALT mildly elevated (ULN < ALT ≤ 3 × ULN, ALT‐ME group), and ALT significantly elevated group (ALT > 3 × ULN, ALT‐SE group). The main analysis was performed between the ALT‐N and ALT‐ME groups.

Univariate logistic regression analysis was performed to assess the association between clinical variables and in‐hospital mortality. All clinical variables with statistical significance (defined as *p* < 0.05) in the univariate analysis, or those with clinical importance, were further assessed in the multivariate analysis using a forward LR method. Multivariate logistic regression was performed using the forward conditional method (forward: conditional), and the results were verified with the forward likelihood ratio method (forward: LR), which produced the same set of variables. A value of *p* < 0.05 (two‐sided) was considered statistically significant in all tests. All data were analyzed using SPSS software 26.0 (SPSS, Inc., Chicago, IL, USA) and R (Version 3.4.3).

## 3. Results

### 3.1. Baseline Characteristics

A total of 2404 patients were diagnosed with AMI in the CCU of Guangdong Provincial People’s Hospital between January 2014 and December 2019. Among these patients, 356 patients were aged ≥ 75 years. From this sample, 19 patients with chronic hepatic disease, 3 with active malignant tumors, and 13 lacking sufficient laboratory tests were excluded; thus, 321 patients were finally included in this study. The baseline clinical characteristics of participants in the different groups based on ALT levels at admission are shown in Table 1. The mean age of the study population was 81 ± 4.1 years, and 63.2% were men. There were no significant differences in the baseline demographic or medical history characteristics between the groups. There were only 16 patients in the ALT‐SE group, and the ALT‐SE group had significantly higher in‐hospital mortality, lower rates of reperfusion therapy, and longer hospital stays than ALT‐N and ALT‐ME groups. Considering the distinguishing clinical features of the patients in the ALT‐SE group, the outcome analysis in this study was between the ALT‐N and ALT‐ME groups.

**Table 1:** Baseline clinical characteristics and in‐hospital mortality of older patients with acute myocardial infarction.

Variable	Overall (*n* = 321)	ALT‐N group ALT ≤ ULN (*n* = 201)	ALT‐ME group ULN < ALT ≤ 3 × ULN (*n* = 104)	*p* value Group 2 vs Group 1	ALT‐SE group ALT > 3 × ULN (*n* = 16)	*p* value Group 3 vs Group 1
Age (years)	81 ± 4.1	81.0 ± 4.2	80.8 ± 3.9	0.888	82.1 ± 3.3	0.136
Male (*n*, %)	203 (63.2)	132 (65.7)	64 (61.5)	0.529	7 (43.8)	0.104
Medical history (*n*, %)						
Smoking	96 (29.9)	60 (29.9)	34 (32.7)	0.604	2 (12.5)	0.163
Diabetes	123 (38.3)	77 (38.3)	39 (37.5)	0.902	7 (43.8)	0.791
Hypertension	211 (65.7)	136 (67.7)	65 (62.5)	0.375	10 (62.5)	0.783
Prior MI, PCI, or CABG	44 (13.7)	32 (15.9)	9 (8.7)	0.110	3 (18.8)	0.727
Clinical characteristics (*n*, %)						
Statin usage at admission	263 (81.9)	197 (98.0)	66 (63.5)	< 0.001	0 (0)	< 0.001
Statin usage at discharge	264 (93.3)	185 (97.4)	73 (86.9)	< 0.001	6 (66.7)	0.003
STEMI	216 (67.3)	127 (63.2)	78 (75.0)	0.040	11 (68.8)	0.790
Killip class ≥ 2	149 (46.4)	86 (42.8)	52 (50.0)	0.275	11 (68.8)	0.065
Right ventricular infarction	18 (5.6)	6 (3.0)	11 (10.6)	0.009	1 (6.3)	0.420
Cardiac arrest before admission	10 (3.1)	3 (1.5)	6 (5.8)	0.067	1 (6.3)	0.266
Heart rate at admission (per minute)	83.7 ± 17.7	83.7 ± 16.7	83.2 ± 18.9	0.836	86.3 ± 21.8	0.197
SBP (mmHg)	125.4 ± 25.0	128.0 ± 24.5	122.8 ± 25.5	0.061	110.1 ± 22.9	0.003
DBP (mmHg)	73.1 ± 15.3	73.6 ± 14.2	73.0 ± 16.9	0.477	67.4 ± 15.9	0.045
Hospital stay (days)	8.0 (6.0–11.0)	8.0 (6.0–10.0)	8.5 (6.0–12.0)	0.018	11.5 (4.3–21.0)	0.103
Revascularization information (*n*, %)						
S2B time within 12 h	136 (42.4)	83 (41.3)	47 (45.2)	0.543	6 (37.5)	1.000
S2B time within 48 h	224 (69.8)	139 (69.2)	78 (75.0)	0.351	7 (43.8)	0.051
PCI	285 (88.8)	182 (90.5)	92 (88.5)	0.556	11 (68.8)	0.021
Multivessel disease	252 (81.0)	161 (81.7)	79 (79.0)	0.640	12 (85.7)	1.000
Biochemical variables						
ALT (U/L)	72.3 ± 184.4	26.7 ± 10.6	76.3 ± 25.3	< 0.001	618.4 ± 610.9	< 0.001
Peak ALT within 72 h (U/L)	123.6 ± 377.7	46.4 ± 211.3	185.9 ± 473.1	< 0.001	704.8 ± 685.1	< 0.001
AST/ALT	3.69 ± 2.35	3.56 ± 2.25	4.24 ± 2.40	0.010	1.80 ± 1.56	< 0.001
GGT (U/L)	47.2 ± 61.8	32.8 ± 27.2	62.5 ± 91.3	< 0.001	98.1 ± 52.1	< 0.001
Total bilirubin (mmol/L)	16.0 ± 7.6	14.8 ± 6.6	17.0 ± 7.22	0.005	24.7 ± 13.2	< 0.001
Albumin (g/L)	34.49 ± 4.03	34.8 ± 3.7	34.5 ± 3.9	0.506	30.3 ± 6.42	0.002
INR	1.16 ± 0.30	1.10 ± 0.13	1.21 ± 0.36	< 0.001	1.62 ± 0.78	< 0.001
Fibrinogen (g/L)	4.61 ± 14.7	4.68 ± 1.42	4.43 ± 1.47	0.103	4.74 ± 2.03	0.920
Peak CK (U/L)	1978.6 ± 1969.5	1459.9 ± 1496.6	2871.2 ± 2322.4	< 0.001	2513.2 ± 2429.9	0.202
Peak CK‐MB (U/L)	192.2 ± 207.8	143.0 ± 138.7	275.4 ± 266.5	< 0.001	262.1 ± 295.1	0.229
Hemoglobin (g/L)	122.1 ± 20.6	122.2 ± 20.0	124.8 ± 20.2	0.506	101.9 ± 21.1	< 0.001
Platelet count (10^9^/L)	215.8 ± 72.5	222.7 ± 75.7	206.9 ± 64.8	0.162	187.1 ± 70.2	0.053
eGFR (mL/min/1.73 m^2^)	66.17 ± 29.85	71.65 ± 30.70	60.78 ± 25.18	0.003	32.3 ± 16.2	< 0.001
Total cholesterol (mmol/L)	4.52 ± 1.04	4.50 ± 1.00	4.68 ± 1.06	0.142	3.89 ± 1.13	0.022
Triglyceride (mmol/L)	1.35 ± 0.72	1.36 ± 0.75	1.36 ± 0.70	0.990	1.11 ± 0.41	0.242
LDL‐C (mmol/L)	2.92 ± 0.80	2.94 ± 0.79	2.94 ± 0.79	0.982	2.52 ± 0.91	0.043
NT‐proBNP (pg/mL)	3728 (1312–9337)	2586 (1053–8241)	4501 (1993–11125)	0.003	14,413 (3868–35,000)	< 0.001
LVEF (%)	47.0 (37.0–58.0)	48.0 (40.0–60.0)	43.5 (35.0–55.8)	0.007	33 (26–51)	0.002
In‐hospital death (%)	37 (11.5)	12 (6.0)	19 (18.3)	0.001	6 (37.5)	< 0.001

Abbreviations: CK, creatine kinase; DBP, diastolic blood pressure; S2B time, symptoms to balloon time; SBP, systolic blood pressure; ULN, upper limit of normal.

Compared to the ALT‐N group, the ALT‐ME group had a significantly lower rate of statin use upon admission (63.5% vs. 98.0%, *p* < 0.001) and discharge (86.9% vs. 97.4%, *p* < 0.001), and higher rates of STEMI (75.0% vs. 63.2%, *p* = 0.040) and right ventricular infarction (10.6% vs. 3.0%, *p* = 0.009). The ALT‐ME group had a lower eGFR level than the ALT‐N group (*p* = 0.003). Hepatic laboratory parameters, including gamma‐glutamyl transferase (GGT, *p* < 0.001), INR derived from prothrombin time (*p* < 0.001), and bilirubin levels (*p* = 0.005), were also significantly different between the groups. Peak creatine kinase‐MB (CK‐MB) levels were significantly higher in the ALT‐ME group (*p* < 0.001), possibly indicating a larger infarction area.

### 3.2. ALT Change and Statin Intolerance

Fifty‐eight patients (18.1%) were considered intolerant to any dose of statins in our study, indicating that statin intolerance is common in older patients with AMI. Patients with statin intolerance upon admission had significantly higher mortality rates than those who started early statin therapy (31.0% vs. 7.2%, *p* < 0.001). Forty statin‐intolerant patients in this study survived AMI, and 26 (65.0%) started statin therapy before discharge.

**Figure 1:**
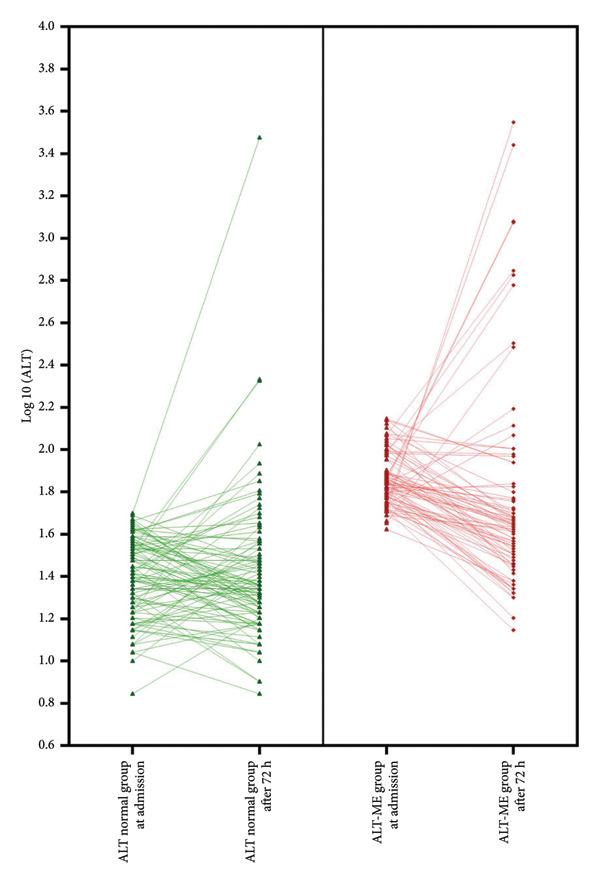
ALT change during hospitalization in ALT‐N (ALT normal) and ALT‐ME (ALT mildly elevated) groups.

**Figure 2:**
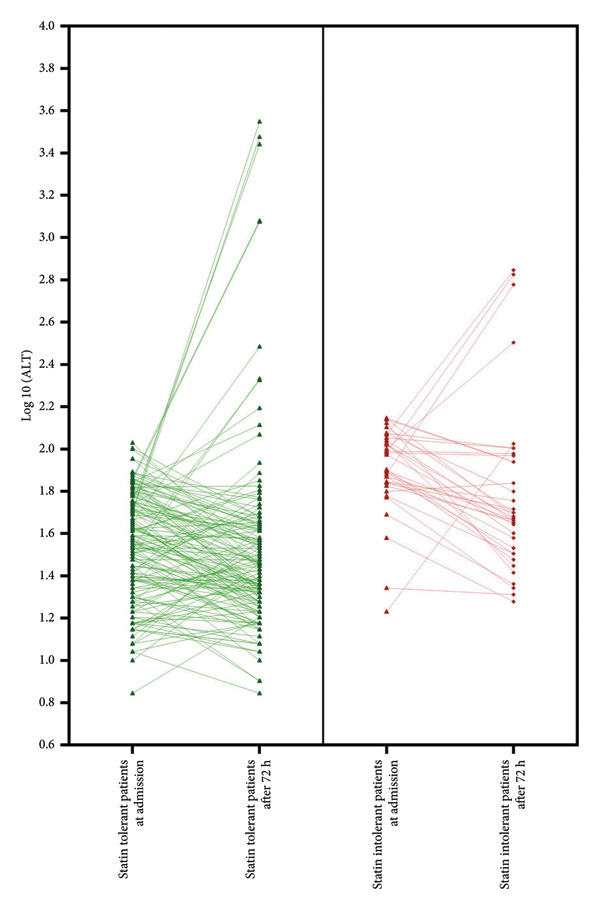
ALT change during hospitalization in patients with or without admission statin intolerance.

The ALT‐N and ALT‐ME groups included 98 (48.8%) and 84 (80.8%) repeated ALT tests, respectively. Figures 1 and 2 demonstrate the trends in ALT levels in participants in the different groups based on ALT levels at admission and statin tolerance states, respectively.

### 3.3. Outcomes

Patients in the ALT‐ME group had a significantly worse clinical prognosis than those in the ALT‐N group. In‐hospital mortality was significantly higher in the ALT‐ME group (18.3% vs. 6.0%, *p* = 0.001), and the incidence of mechanical complications of AMI, cardiogenic shock, severe ventricular arrhythmia, and bleeding events are also significantly higher in the ALT‐ME group (Table 2). Patients in the ALT‐ME group had a lower rate of ischemic stroke than those in the ALT‐N group, probably towing to prolonged coagulation time.

**Table 2:** In‐hospital outcomes in ALT‐N and ALT‐ME groups.

In‐hospital Outcomes	ALT‐N group ALT ≤ ULN (*n* = 201)	ALT‐ME group ULN < ALT ≤ 3 × ULN (*n* = 104)	*p* value
All‐cause death	12 (6.0%)	19 (18.3%)	0.001
Mechanical complications of AMI	4 (2.0%)	7 (6.7%)	0.050
Cardiogenic shock	21 (10.4%)	25 (24.0%)	0.002
Severe ventricular arrhythmia	4 (2.8%)	8 (7.7%)	0.026
Ischemic stroke	9 (4.5%)	1 (1.0%)	0.173
Bleeding events	27 (13.4%)	20 (19.2%)	0.184
Major bleeding	7 (3.5%)	6 (5.8%)	0.378
Minor bleeding	3 (1.5%)	4 (3.5%)	0.235
Minimal bleeding	17 (8.5%)	10 (9.6%)	0.736
Coagulopathy	4 (2.0%)	16 (15.4%)	< 0.001
In‐hospital ALT elevation	6 (4.2%)	12 (12.2%)	0.019

*Note:* ALT, alanine aminotransferase.

Abbreviations: ALT‐ME, ALT mildly elevated; ALT‐N, ALT normal; AMI, acute myocardial infarction.

### 3.4. Logistic Analysis

Logistic analysis was performed in the ALT‐N and ALT‐ME groups to confirm the association between ALT levels upon admission and in‐hospital mortality. In the univariate logistic analysis, Killip class, cardiac arrest before admission, admission systolic blood pressure, admission ALT, peak CK‐MB, eGFR, N‐terminal probrain natriuretic peptide, and LVEF significantly predicted in‐hospital mortality (per 10 U/L, odds ratio [OR] 1.250, confidence interval [CI] 1.121–1.394, *p* < 0.001; Table 3). In multivariate logistic analysis, after adjusting for potential confounding factors including age, STEMI, early revascularization within 48 h, and the above potential confounders, ALT levels upon admission remained independently associated with in‐hospital mortality (per 10 U/L, OR 1.164, CI 1.037–1.305, *p* = 0.010, Table 3).

**Table 3:** Univariate and multivariate logistic regression analyses for in‐hospital mortality.

Variable	Univariate analysis		Multivariate analysis	
	Odd ratios (95% CI)	*p* value	Odd ratios (95% CI)	*p* value
Age	1.038 (0.951–1.132)	0.401		
Sex: Female	0.747 (0.351–1.589)	0.449		
Diabetes	1.567 (0.695–3.534)	0.279		
STEMI	1.762 (0.732–4.240)	0.206	2.907 (1.058–7.985)	0.039
Killip class ≥ 2	5.937 (2.359–14.940)	< 0.001	5.185 (1.976–13.604)	0.001
Right ventricular infarction	1.990 (0.539–7.348)	0.302		
Cardiac arrest before admission	7.970 (2.019–31.461)	0.003		
SBP (per 10 mmHg)	0.856 (0.730–1.004)	0.056		
Early revascularization within 48 h	0.608 (0.282–1.312)	0.205		
Multivessel disease	1.156 (0.421–3.173)	0.779		
ALT (per 10 U/L)	1.250 (1.121–1.394)	< 0.001	1.164 (1.037–1.305)	0.010
Peak CK‐MB (per 10 U/L)	1.013 (0.998–1.029)	0.089		
Hemoglobin (per g/L)	0.988 (0.971–1.006)	0.192		
eGFR (per 10 mL/min/1.73 m^2^)	0.792 (0.685–0.914)	< 0.001	0.812 (0.683–0.966)	0.019
Lg NT‐proBNP	4.157 (1.897–9.109)	< 0.001		
LVEF (per %)	0.960 (0.932–0.989)	0.008		

*Note:* A forward LR regression method was used in the multivariate logistic analysis, adjusted for STEMI, Killip class, cardiac arrest before admission, systolic blood pressure, peak CK‐MB, eGFR, Lg NT‐proBNP, and LVEF. STEMI, ST‐segment elevation myocardial infarction; ALT, alanine aminotransferase; CK‐MB, creatine kinase isoenzyme MB.

Abbreviations: CI, confidence interval; CK, creatine kinase; DBP, diastolic blood pressure; eGFR, estimated glomerular filtration rate; LVEF, left ventricular ejection fraction; NT‐proBNP, N‐terminal probrain natriuretic peptide; S2B time, symptoms to balloon time; SBP, systolic blood pressure; ULN, upper limit of normal.

## 4. Discussion

In this retrospective cohort study, we found that ALT levels upon admission, even when mildly elevated, were independently associated with in‐hospital mortality and other AMI‐related adverse outcomes in older patients with AMI. In our study, 18.1% of older patients with AMI upon admission and 6.7% discharge were considered statin intolerant, which was much higher than the reported prevalence in the general AMI population [21]. Elevated ALT levels upon admission in most patients recovered to normal, and statin therapy was initiated in most older AMI survivors upon discharge. Compared with similar retrospective studies on ALT elevation in patients with AMI, our study focused on older patients aged ≥ 75 years, who are more vulnerable to ischemic attack, and our study analyzed the dynamic change in ALT level during hospitalization and its relation with statin therapy. Elderly patients are more susceptible to hepatic dysfunction, hemodynamic instability, and statin intolerance than younger AMI populations, making their clinical management substantially different [22]. Previous studies have rarely examined in‐hospital ALT dynamics or evaluated how transient ALT fluctuations affect statin use specifically in patients aged ≥ 75 years [23–25]. Our findings therefore complement existing research by addressing this evidence gap and providing clinically relevant information for this high‐risk group. Based on our findings, we recommend that liver function tests, including ALT, be routinely assessed in elderly patients or those at high risk of hepatic impairment upon admission for acute MI. If ALT elevation is observed during hospitalization, repeat testing before discharge is advisable. This approach allows for better evaluation of statin tolerance and informed selection of long‐term lipid‐lowering therapy.

It has been discovered that ALT levels can be significantly elevated in some patients with AMI; however, the underlying clinical implications remain unclear [8, 26–29]. Unlike earlier studies that primarily assessed baseline ALT levels, our work demonstrates that even mild or transient ALT elevations during hospitalization have prognostic value in older AMI patients. This dynamic assessment provides a more sensitive reflection of hepatic stress and cardiovascular instability in the elderly. ALT and AST are the two main aminotransferases that are widely used in clinical laboratory tests. AST, which is enriched in myocardial tissue, has long been considered a cardiac enzyme used in AMI diagnosis, and its fluctuation pattern after AMI is stable [30]. ALT is also an enzyme found in myocardial tissue; however, significant ALT elevation is not typical after AMI and is considered to be related to hepatic dysfunction [28]. Although ALT and AST elevation correlate with peak CK‐MB level, which is a key indicator of the myocardial infarction area, peak ALT levels in more than half of patients with AMI are within the normal range, whereas AST is significantly elevated in most patients with AMI.

Although many retrospective studies have confirmed that ALT elevation is associated with a worse prognosis in patients with AMI [8, 13, 31], the underlying role of ALT levels in AMI is unclear. Some studies have suggested that elevated serum ALT is merely a result of more extensive myocardial injury but not an indicator of liver damage [13, 27], whereas others have concluded that ALT elevation is a biomarker of liver damage, primarily caused by heart failure in patients with AMI [9, 32]. Most cardiovascular studies use 3 × ULN as the cutoff value for liver dysfunction diagnosis, which is consistent with the blood cholesterol management guidelines [15, 33]. In our study, the prognosis and hepatic function of patients in the ALT‐SE group were worse than the ALT‐N group and the ALT‐ME group. Compared to the ALT‐N group, the liver‐related laboratory test results were significantly worse in the ALT‐ME group, including higher GGT levels (*p* < 0.001), total bilirubin levels (*p* = 0.005), and INR (*p* < 0.001). These findings were consistent with those of a recent study [33]. In elderly patients, ALT elevation may carry stronger prognostic significance because this population is more prone to hemodynamic instability, multiorgan vulnerability, and impaired physiological reserve, making even mild hepatic injury a marker of substantial systemic stress. The proportion of patients with heart failure was also higher in the ALT‐ME group than in the ALT‐N group. Considering that heart failure is common in patients with AMI and usually causes congestive or ischemic liver damage [34–36], the results of our study indicate that mildly elevated ALT levels could be a result of mild liver injury caused by heart failure. Some population‐based cohort studies on the general population have suggested that elevated liver enzymes are associated with higher cardiovascular disease risk and mortality [37, 38], in which nonalcoholic fatty liver disease and hyperlipidemia may be the underlying mechanisms. This situation is common in older patients with AMI and should be considered. However, 87.4% of the AMI survivors with elevated ALT in our study had ALT levels that recovered to the normal range when discharged, making it unlikely to be a chronic process. In our study, multivariate analysis confirmed that ALT elevation was independently associated with in‐hospital mortality after adjusting for CK‐MB levels, which further supported that mild ALT elevation is not a simple result of a larger infarction area and is rather a potential risk stratification parameter reflecting different pathophysiological situations. In addition to heart failure, right ventricular infarction may also contribute to liver dysfunction. In our study, the incidence of right ventricular infarction was significantly higher in the ALT‐ME group compared to the ALT‐N group (10.6% vs. 3.0%, *p* = 0.009). Although not independently associated with mortality, right ventricular infarction can lead to elevated central venous pressure and hepatic congestion, contributing to ALT elevation. Furthermore, cardiogenic shock, which was more frequent in the ALT‐ME group, can lead to hepatic hypoperfusion and ischemic liver injury. A recent cardiac MRI study in STEMI patients found that early hepatic T1 relaxation times correlated with both right ventricular infarction and infarct size and were influenced by hepatic fat and systemic inflammation [39]. These findings suggest that a combination of hepatic ischemia, venous congestion, inflammation, and steatosis may underlie the observed ALT elevation.

Another effect of elevated ALT levels in patients with AMI is its influence on statin therapy. Statin therapy remains the cornerstone of AMI management in the current guidelines, and statins are indicated in all patients with AMI to improve their prognosis [2, 40, 41]. The abnormal transaminase test is still considered one of the statin associated side effects on liver, and withdrawal of statin therapy is recommended when ALT elevates > 3 × ULN [15, 16]. A population‐based study indicated that discontinuation of statins in AMI survivors is relatively rare [42]; however, the results of our study suggested that statin intolerance in older patients with AMI may be more common than anticipated. In our study, the main reason against statin prescription upon admission was significant ALT elevation or concerns of further liver damage, and only six patients had a history of statin‐associated muscle symptoms as a statin contraindication before admission. No patients in the ALT‐SE group started statin therapy upon admission. Patients in the ALT‐ME group who could not tolerate statin therapy at admission still had higher mortality rates than the other patients in this group (28.1% vs. 12.4%, *p* = 0.053). Older patients may have been underrepresented in previous studies, and our study highlighted that the statin intolerance rate was higher in older Asian patients with AMI. A retrospective study from China including 2417 STEMI patients (mean age 59.5 years) also reported a 5.0% incidence of ALT ≥ 125 U/L [12]. In the PROVE IT–TIMI 22 trial, which enrolled 4162 ACS patients (mean age 58), ALT elevations ≥ 3 × ULN occurred in 1.1% and 3.3% of the moderate‐ and high‐intensity statin groups, respectively [43]. Similarly, a meta‐analysis of 12 trials on early statin use found higher ALT elevations in statin‐treated patients (1.1%) compared to controls (0.4%) [44]. These findings suggest that both the severity of ACS and statin exposure contribute to ALT elevation. The results of a recent meta‐analysis also support the fact that the prevalence of statin intolerance is higher in specific patient groups [45].

Our findings indicated that older patients with AMI and elevated baseline ALT levels are at higher risk of adverse events and statin intolerance, whereas high‐risk patients may benefit more from early lipid control [46]. Evolving lipid‐lowering agents such as ezetimibe and PCSK9 inhibitors have been proven to be effective in LDL‐C control [47, 48], and these medications should be considered in older patients with AMI with abnormal liver enzyme results. A recent single‐center real‐world study in China also showed that PCSK9 inhibitors are effective and safe for short‐term lipid control in patients with AMI [49]. Considering that most patients with elevated ALT levels upon admission can receive statin therapy when discharged, short‐term PCSK9 inhibitor use upon admission could be a promising solution for these patients.

## 5. Conclusion

Our study had some limitations. First, as a retrospective study, some participants, mainly those with normal ALT levels upon admission, did not repeat the ALT test during their hospital stay. Missing information from patients who did not undergo repeat ALT laboratory tests or died within 3 days after admission hindered further analysis of ALT trends in our study population. Second, statin usage decisions in our study relied on the physicians’ clinical judgment, and not on a fixed standard. This method still fulfilled the international definition; however, it prevented us from obtaining a more exact prevalence of statin intolerance in this study. Third, we only evaluated the relationship between elevated ALT levels and in‐hospital outcomes. Because long‐term follow‐up data were unavailable, we could not determine whether transient ALT elevation during AMI has a sustained effect on long‐term prognosis. Future prospective studies with systematic follow‐up will provide a more comprehensive assessment of the impact of transient ALT elevation during AMI on long‐term prognosis. Fourth, the definition and adjudication of statin intolerance in this study were limited by its retrospective nature. Clinical decisions were made between 2014 and 2019 and were based on the guidelines available at that time, which have since evolved. Moreover, creatine kinase (CK) levels were commonly elevated due to AMI itself, limiting the utility of this marker for diagnosing muscle‐related statin side effects. Consequently, assessment relied heavily on physician documentation, symptom history, and clinical judgment. To reduce misclassification, we adopted a strict definition of complete statin intolerance, which may have underestimated the true prevalence of intolerance in this population. Finally, as a retrospective study, this analysis cannot establish a causal relationship between elevated ALT levels, in‐hospital mortality, and statin intolerance. Further prospective studies are needed to validate and expand upon these findings.

Our study is still of critical clinical importance. It focused on older patients with AMI, a patient group underrepresented in previous studies on liver dysfunction and statin intolerance. It investigated ALT trends and statin usage from admission to discharge, which have rarely been documented in previous studies. Our study provides additional information about statin intolerance epidemiology and indicated that new lipid‐lowering agents, such as PCSK9 inhibitors, may be an ideal option for older patients with AMI, especially those with liver dysfunction. Further prospective studies are needed to explore the role of ALT in patients with AMI and optimize lipid control algorithms in these patients.

In conclusion, even mildly elevated ALT levels upon admission are simple and strong predictors of adverse in‐hospital outcomes in older patients with AMI. Statin intolerance is more common than anticipated in older patients with AMI during the acute phase.

**Nomenclature**
ALT: Alanine transaminase
AMI: Acute myocardial infarction
ULN: Upper limit of normal
ALT‐N: ALT normal
ALT‐ME: ALT mildly elevated
GGT: Gamma‐glutamyl transferase
INR: International normalized ratio
CAD: Coronary artery disease
LDL‐C: Low‐density lipoprotein cholesterol
PCSK9: Proprotein convertase subtilisin/kexin type 9
CCU: Cardiac care unit
STEMI: ST‐segment elevation myocardial infarction
NSTEMI: Non‐ST‐segment elevation myocardial infarction
PCI: Percutaneous coronary intervention
eGFR: Estimated glomerular filtration rate
CK‐MB: Creatine kinase isoenzyme‐MB
NT‐proBNP: N‐terminal probrain natriuretic peptide
LVEF: Left ventricular ejection fraction
AST: Aspartate aminotransferase
SBP: Systolic blood pressure
DBP: Diastolic blood pressure
S2B time: Symptoms to balloon time
CK: Creatine kinase
ULN: Upper limit of normal

## Conflicts of Interest

The authors declare no conflicts of interest.

## Funding

The authors received no specific funding for this work.

## Data Availability

The data that support the findings of this study are available from the corresponding author upon reasonable request.
